# Clinical study of remimazolam combined with ultrasound-guided superior laryngeal nerve block in elderly patients with coronary heart disease undergoing bronchoscopy

**DOI:** 10.3389/fmed.2026.1840825

**Published:** 2026-07-30

**Authors:** Yuqian Sang, Yanan Wu, Huifang Bai, Ling Yang

**Affiliations:** 1 School of Anesthesiology, Shanxi Medical University, Taiyuan, China; 2 Department of Anesthesiology, First Hospital of Shanxi Medical University, Taiyuan, China

**Keywords:** bronchoscopy, coronary heart disease, elderly patients, remimazolam, superior laryngeal nerve block

## Abstract

**Purpose:**

To investigate the efficacy and safety of remimazolam combined with ultrasound-guided superior laryngeal nerve block (SLNB) during bronchoscopic procedures in elderly patients with coronary heart disease.

**Methods:**

A total of 99 elderly patients with coronary heart disease who underwent a flexible bronchoscopy procedure at the First Hospital of Shanxi Medical University from March 2025 to August 2025 were enrolled and randomly divided into three groups (*n* = 33 each): Group R (remimazolam plus topical anesthesia), Group RSL (remimazolam plus topical anesthesia plus SLNB), and Group PSL (propofol plus topical anesthesia plus SLNB). Hemodynamic parameters (MAP, HR, SpO_2_) were recorded at five time points: before oxygen inhalation after entering the operating room (T0), after anesthesia induction (T1), bronchoscope passage through the vocal cords (T2), at the end of the procedure (T3), and immediately upon leaving the operating room (T4). Cognitive function (MMSE, MoCA, SAS, SDS) was assessed at three time points: 1 h preoperatively (d0), 1 day postoperatively (d1), and 7 days postoperatively (d7). Inflammatory and stress markers (TNF-α, CRP, and COR) were measured at two time points: preoperatively (T0) and upon leaving the operating room (T4). Anesthesia outcomes and adverse events were also compared. Longitudinal outcomes were analyzed using linear mixed-effects models, with repeated-measures ANOVA performed as a sensitivity analysis.

**Results:**

No significant differences were observed in baseline characteristics among the three groups (*P* > 0.05). Hemodynamic parameters showed no significant differences at T0, T1, or T4 (*P* > 0.05). However, at T2 and T3, MAP and HR in Group R were significantly higher than those in Group RSL and Group PSL (*P* < 0.001), while SpO_2_ was significantly lower (*P* < 0.001). No significant differences in MAP or HR were found between Group RSL and Group PSL at T2 or T3 (*P* > 0.05), but SpO_2_ was higher in Group RSL than in Group PSL (T2: *P* = 0.003; T3: *P* = 0.004). Regarding cognitive function, no significant differences were observed between Group R and Group PSL at any time point (*P* > 0.05). At d1, MMSE and MoCA scores in Group RSL were higher than those in Group R and Group PSL (*F* = 6.54, *P* = 0.002; *F* = 7.51, *P* = 0.001), while SAS and SDS scores were lower (*F* = 6.69, *P* = 0.002; *F* = 4.64, *P* = 0.012). CRP levels showed no significant differences across groups at any time point (*P* > 0.05). At T4, TNF-α and COR levels differed significantly among the three groups (*F* = 8.75, *P* < 0.001; *F* = 5.16, *P* = 0.007), with Group R showing significantly higher levels than Group RSL and Group PSL (*P* < 0.05), whereas no significant differences were detected between Group RSL and Group PSL (*P* > 0.05). In terms of anesthesia outcomes, Group RSL and Group PSL had a higher proportion of vocal cord opening, greater patient and endoscopist satisfaction (*P* < 0.001, *P* = 0.015), shorter recovery time (*F* = 4.64, *P* = 0.010), and lower intraoperative remifentanil consumption (*F* = 35.42, *P* < 0.001) compared with Group R. Recovery time was shorter in Group RSL than in Group PSL. However, the difference was not statistically significant (*P* > 0.05). Regarding adverse events, the incidence of intraoperative coughing, hypoxemia, and postoperative nausea and vomiting was significantly higher in Group R than in Group RSL and Group PSL (*P* < 0.05). Group RSL had a lower incidence of intraoperative hypoxemia than Group PSL (*P* = 0.046). No block-related complications were observed in Group RSL or Group PSL.

**Discussion:**

Ultrasound-guided superior laryngeal nerve block combined with remimazolam for bronchoscopy in elderly patients with coronary heart disease helps maintain hemodynamic stability, suppresses coughing, reduces adverse reactions, shortens recovery time, lowers remifentanil consumption and hypoxemia incidence, and enhances anesthesia satisfaction, suggesting that this regimen may be a feasible option for bronchoscopy sedation. Although transient differences in early postoperative cognitive screening scores were observed, their clinical significance remains unclear and warrants cautious interpretation.

## 1 Introduction

Bronchoscopy is widely used for diagnosing and treating lung diseases due to its convenience and efficiency. However, the procedure is invasive, may cause hypoxemia, and often results in significant patient discomfort. As the population ages, more elderly patients with coronary heart disease are undergoing bronchoscopy. This group is more susceptible to hemodynamic fluctuations, which can cause cardiac and cerebral ischemia and hypoxia, increasing perioperative cardiovascular and cerebrovascular risk. Additionally, elderly patients often experience cognitive decline, slower recovery, and more complications postoperatively.

Remimazolam takes effect rapidly, is rapidly metabolized, and has minimal effects on respiratory and circulatory functions, making it particularly suitable for elderly patients (1, 2). Ultrasound-guided superior laryngeal nerve block (SLNB) can precisely block sensation in the larynx, effectively suppressing the gag reflex (3). In addition, SLNB may reduce sedative and opioid requirements and contribute to improved hemodynamic stability. To date, no reports have described the combined use of these two techniques during bronchoscopy in elderly patients with coronary heart disease. This study aimed to investigate the efficacy and safety of this combination regimen to provide clinicians with an optimized anesthesia strategy.

## 2 Materials and methods

### 2.1 Ethics and registration

The Ethics Committee of the First Hospital of Shanxi Medical University approved this study on January 21, 2025 (No. KYLL-2025-002). All procedures followed the institution’s Research Ethics Committee standards and the Declaration of Helsinki. The study was registered with the China Clinical Trials Registry (ChiCTR2600121168). Investigators explained the study to all subjects, who then signed informed consent forms.

### 2.2 Subjects

**FIGURE 1:**
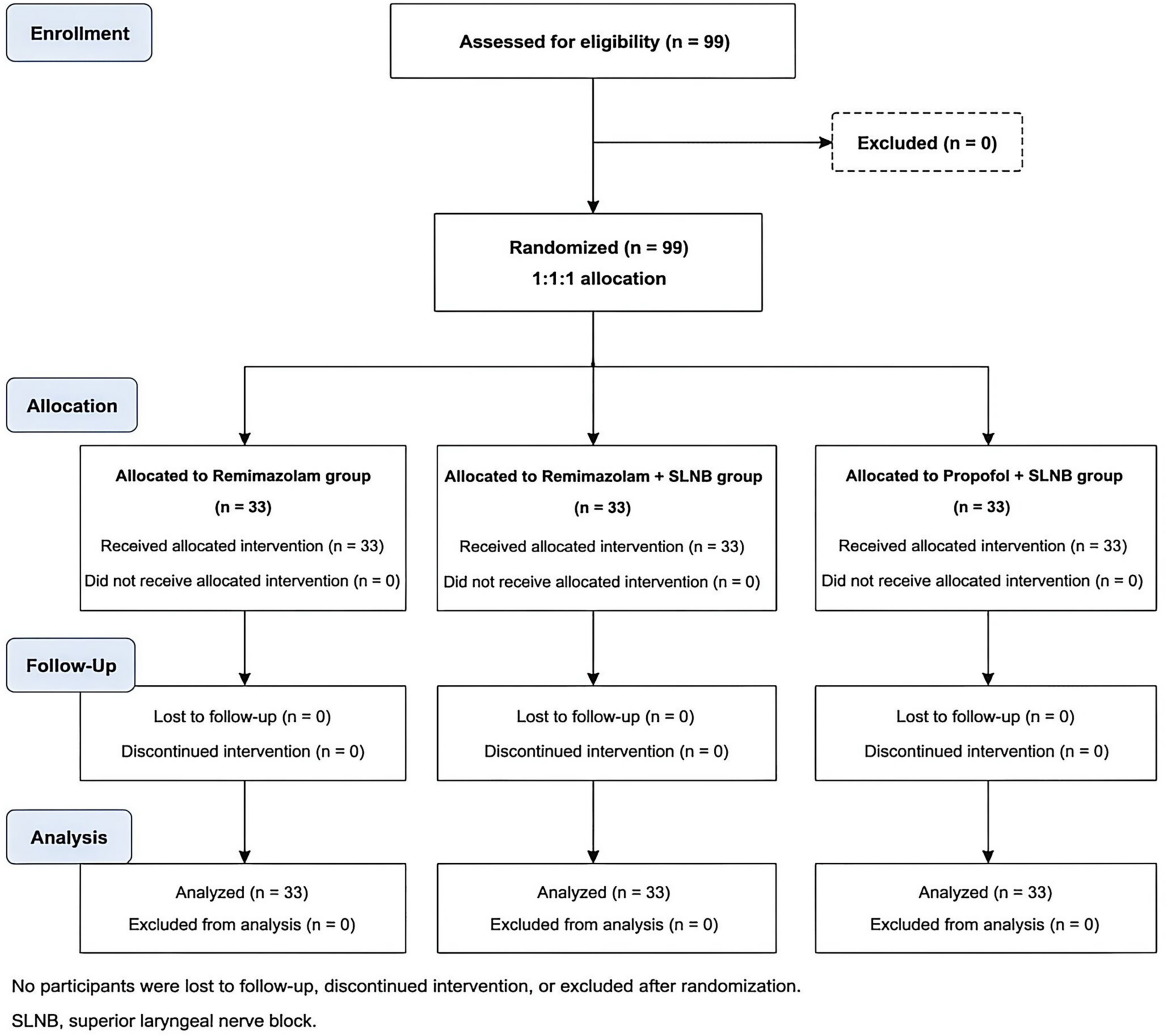
CONSORT flow diagram.

We selected 99 elderly patients (ages 60–80) with coronary heart disease who underwent a flexible bronchoscopy procedure at the First Hospital of Shanxi Medical University from March to August 2025. Patients met ASA physical status II or III, with sex unrestricted. The participant flow throughout the trial is shown in Figure 1.

#### 2.2.1 Inclusion criteria

(1) Diagnosis of coronary heart disease (WHO: ≥50% stenosis in a major coronary artery or branch on angiography); (2) Good mental and cognitive function; (3) Informed consent from patient and family; (4) Well-controlled, stable coronary heart disease and NYHA Class I cardiac function.

#### 2.2.2 Exclusion criteria

(1) Other organic heart disease; (2) Heart failure, arrhythmias, or similar conditions; (3) History of severe cardiovascular or cerebrovascular events (such as myocardial or cerebral infarction); (4) Cardiac or cranial surgery history; (5) Allergies to benzodiazepines or local anesthetics; (6) History of laryngeal surgery, infectious neck wounds, upper respiratory infection, chronic pharyngitis, or sore throat; (7) Oral anatomical abnormalities or pre-exam hoarseness; (8) Cognitive impairments including alcohol/drug abuse, epilepsy, or psychiatric disorders; (9) Patients with coagulation disorders.

#### 2.2.3 Randomization and blind methods

Patients were randomly assigned in a 1:1:1 ratio to the three groups using a random number table: Group R, which received remimazolam and topical anesthesia; Group RSL, which received remimazolam, ultrasound-guided superior laryngeal nerve block (SLNB), and topical anesthesia; or Group PSL, which received propofol, SLNB, and topical anesthesia. The randomization sequence was generated and maintained by an independent researcher who was not involved in patient enrollment, patient management, outcome assessment, or data analysis. Investigators responsible for participant enrollment did not have access to the allocation sequence. Group assignments were revealed only after participant enrollment and baseline assessment had been completed, thereby maintaining allocation concealment. The protocol for topical lidocaine administration was standardized across all groups. All procedures were performed by experienced bronchoscopists from the same clinical team. On the day of surgery, a researcher who was aware of the assignments instructed the pre-anesthesia room anesthesiologist to administer the nerve block to patients in the RSL and PSL groups. All patients were prepared in the pre-anesthesia room. Other research team members, including perioperative anesthesiologists, surgeons, nurses, and data collectors, remained blinded to group assignments. Investigators responsible for postoperative cognitive assessments were blinded to group allocation.

### 2.3 Anesthesia methods

**FIGURE 2:**
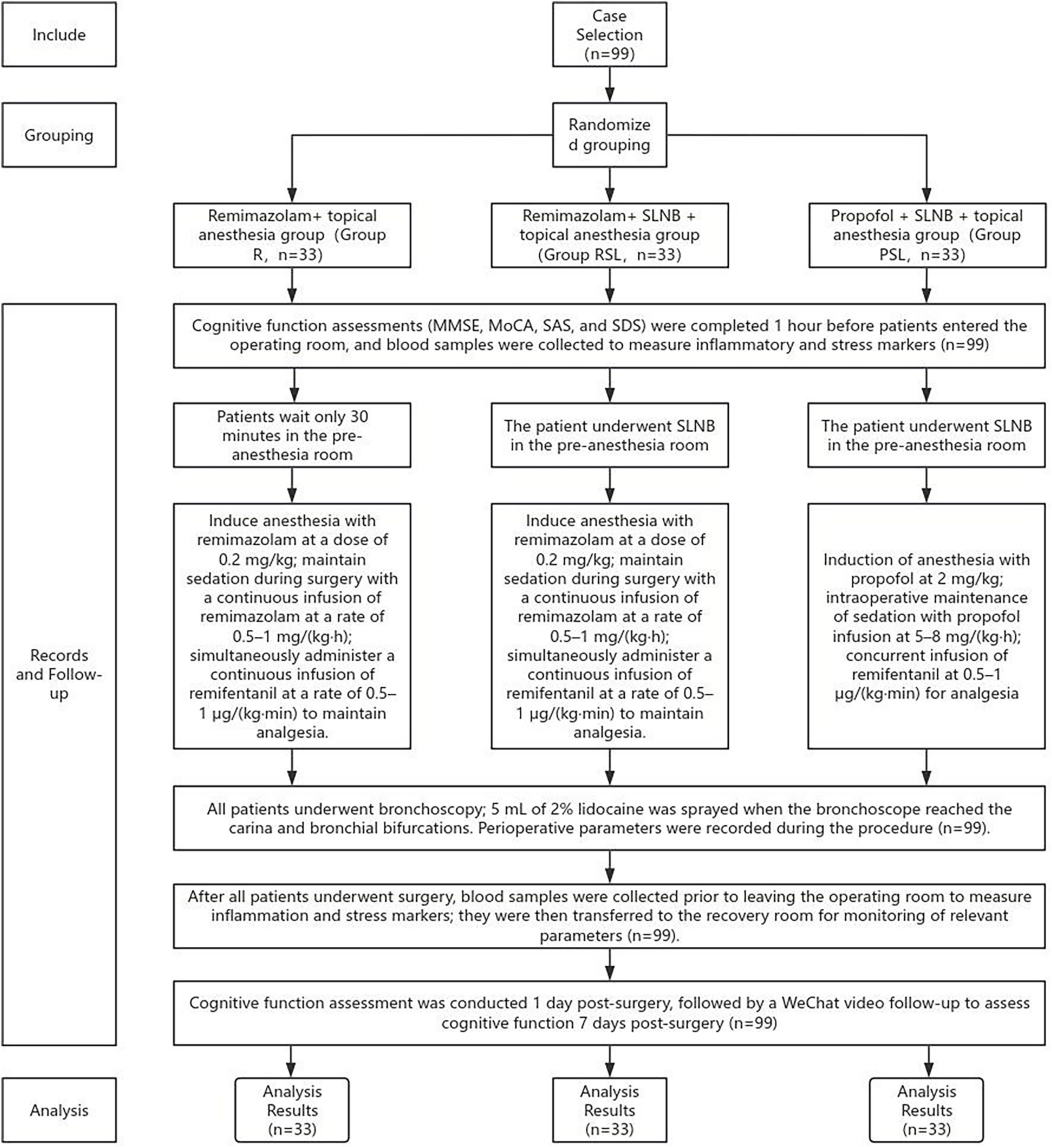
Study protocol of the randomized controlled trial. Patients were randomly assigned to three groups: remimazolam plus topical anesthesia (Group R), remimazolam combined with ultrasound-guided superior laryngeal nerve block (Group RSL), and propofol combined with ultrasound-guided superior laryngeal nerve block (Group PSL). The figure illustrates the perioperative assessment schedule, anesthesia procedures, postoperative evaluations, and study timeline.

The overall study protocol is shown in Figure 2.

#### 2.3.1 Preoperative preparation

All patients were instructed to fast for 8 h and refrain from drinking for 4 h before surgery. Smoking was prohibited. Upon entering the operating room, a venous line was first established, followed by the initiation of continuous monitoring, including ECG, heart rate, mean arterial pressure, SpO_2_, and BIS. The anesthesia machine was then set to manual ventilation in 100% oxygen at a flow rate of 5 L/min.

#### 2.3.2 Anesthesia procedure

Group R: Anesthesia was induced with an intravenous bolus of remimazolam at a dose of 0.2 mg/kg. Flexible bronchoscopy was performed once the patient’s eyelash reflex had disappeared and the patient no longer responds to verbal stimuli. When the bronchoscope reaches the carina and bronchial bifurcation, 5 mL of 2% lidocaine was sprayed. During the procedure, remimazolam was continuously infused at 0.5–1 mg/(kg⋅h) and remifentanil at 0.5–1 μg/(kg⋅min) until the procedure was completed. BIS was maintained at approximately 50–60 throughout the procedure.

Group RSL: Before surgery, patients received an ultrasound-guided superior laryngeal nerve block (see below for specific procedure); the remainder of the procedure was the same as in Group R.

Group PSL: Propofol combined with SLNB was selected as a comparator because it is commonly used for bronchoscopy sedation in clinical practice. Following an ultrasound-guided superior laryngeal nerve block before surgery, propofol (2 mg/kg) was administered intravenously to induce anesthesia. After the patient’s eyelash reflex had disappeared and there was no response to verbal stimuli, flexible bronchoscopy was performed. When the scope reached the carina and bronchial bifurcation, 5 mL of 2% lidocaine was immediately sprayed. Throughout the procedure, propofol was infused at 5–8 mg/(kg⋅h) and remifentanil at 0.5–1 μg/(kg⋅min) via an infusion pump until completion, with infusion rates adjusted according to clinical responses and BIS monitoring. The BIS score was maintained approximately 50–60 during the procedure.

#### 2.3.3 Ultrasound-guided bilateral superior laryngeal nerve block

The patient was positioned in the supine position with the head tilted backward to fully extend the neck, and standard disinfection and draping were performed. A high-frequency linear array ultrasound transducer was placed transversely in the midline of the anterior neck, with its long axis perpendicular to the patient’s coronal plane. First, the area was scanned between the mandible and the thyroid cartilage to identify the transverse section of the hyoid bone, which appears as an “arched” hyperechoic structure. Using this as an anatomical landmark, the probe was moved slightly outward and downward; a hyperechoic region corresponding to the thyrohyoid membrane will be visible below the hyoid bone. A cross-section of the internal branch of the superior laryngeal nerve was visible on the deep surface of the membrane and medial to the superior laryngeal artery. A puncture was then performed using an in-plane needle insertion technique; after confirming the absence of blood on aspiration, 2 mL of 2% lidocaine solution is slowly injected, with close observation of the spread of the solution to ensure adequate block. The internal branch of the contralateral superior laryngeal nerve is blocked using the same method.

#### 2.3.4 Intraoperative management

We recorded the patient’s heart rate, blood pressure, and oxygen saturation in the preparation room as baseline values. Blood pressure was measured every 3 min during the procedure. If the patient’s mean arterial pressure fell below 30% of baseline, this was considered hypotension; this was recorded and 3 mg of ephedrine was administered. If the patient’s heart rate was less than 50 beats per minute, this was considered sinus bradycardia; 0.5 mg of atropine was administered. If hypotension was present concurrently, 3 mg of ephedrine was administered. During the examination, if the patient’s pulse oxygen saturation fell below 92%, hypoxemia was diagnosed; this was recorded, along with the lowest pulse oxygen saturation value. If the patient’s pulse oximetry fell below 92%, the anesthesiologist implemented interventions such as mandibular thrust and mask-assisted positive-pressure ventilation. No patients required tracheal intubation or advanced airway management, and no procedures were terminated because of respiratory events.

### 2.4 Performance indicators

#### 2.4.1 Intraoperative hemodynamic parameters

Mean arterial pressure (MAP), heart rate (HR), and pulse oxygen saturation (SpO_2_) were recorded at the following time points: upon admission to the operating room without oxygen supplementation (T0), after induction of anesthesia (T1), bronchoscope passage through the vocal cords (T2), at the end of surgery (T3), and immediately before leaving the operating room (T4).

#### 2.4.2 Cognitive function assessment

Cognitive function was assessed 1 h before surgery (d0), 1 day after (d1), and 7 days after (d7), using the Mini-Mental State Examination (MMSE) and Montreal Cognitive Assessment (MoCA). Emotional status was evaluated using the Self-Rating Anxiety Scale (SAS) and Self-Rating Depression Scale (SDS).

#### 2.4.3 Measurement of inflammatory markers and pain mediators

Serum levels of tumor necrosis factor-α (TNF-α), C-reactive protein (CRP), and plasma cortisol (COR) were measured at T0 and T4.

#### 2.4.4 Comparison of anesthetic effects

We recorded time to recovery, defined as the interval from the end of the procedure until the patient regained consciousness, achieved a BIS score greater than 90, and could respond appropriately and follow instructions. We also recorded remifentanil consumption and vocal cord movement. The endoscopist evaluated vocal cord movement before bronchoscope passage through the glottis, classifying it as open, moving, or closed. After the procedure, both the endoscopist and the patient assessed their satisfaction with the anesthetic outcome.

#### 2.4.5 Record the incidence of adverse events

Adverse events were recorded during the procedure and immediate postoperative recovery. These included coughing, body movements during surgery, hypoxemia (SpO_2_ < 92%), and postoperative nausea and vomiting. No serious events required tracheal intubation, procedure termination, or intensive care admission.

### 2.5 Sample size calculation

The sample size was calculated using PASS 2021, with mean arterial pressure (MAP) as the primary outcome. Pilot results showed MAPs of 107.00 ± 8.30 mmHg (R group), 96.50 ± 6.00 mmHg (RSL group), and 89.00 ± 7.20 mmHg (PSL group) during bronchoscope passage through the glottis. With α = 0.05 (two-sided), 1-β = 0.9, and a 20% dropout rate, the final sample size was 33 subjects per group. Rather than relying on repeated-measures design assumptions, the calculation centered on the between-group MAP difference at T2, considered the most clinically relevant time point. Consequently, repeated longitudinal and secondary endpoint analyses were deemed exploratory.

### 2.6 Statistical analysis

Statistical analysis used SPSS 26.0. Normality was tested with the Shapiro–Wilk test, and variance with Levene’s test. Continuous variables are presented as mean ± SD or median (IQR). Group comparisons used one-way ANOVA or Kruskal–Wallis tests, with Bonferroni or Dunn–Bonferroni corrections. Categorical variables were compared using the chi-square or Fisher’s exact test.

Linear mixed-effects models analyzed repeated outcomes, with group, time, and group × time interaction as fixed effects, and participant as a random intercept. Sensitivity analysis used repeated-measures ANOVA, applying Mauchly’s test for sphericity and the Greenhouse–Geisser correction as required. Partial η^2^ was reported. Secondary outcomes were exploratory and were not adjusted for multiplicity. Significance was set at *P* < 0.05 (two-sided). All randomized participants completed the study without loss to follow-up or post-randomization exclusion, so the intention-to-treat and per-protocol populations were identical.

## 3 Results

### 3.1 Comparison of general patient characteristics

There were no statistically significant differences in age, weight, gender, or ASA classification among the three patient groups (*p* > 0.05). See Table 1.

**TABLE 1:** Comparison of general patient characteristics (*n* = 33).

		R	RSL	PSL	*P*-value
Age (years)		69.79 ± 3.39	72.12 ± 5.36	70.79 ± 4.70	0.232
Weight (kg)		64.21 ± 11.37	61.98 ± 13.06	62.21 ± 11.51	0.710
Gender	Male	29 (87.9%)	28 (84.8%)	25 (75.8%)	0.397
	Female	4 (12.1%)	5 (15.2%)	8 (24.2%)	
ASA class	II	17 (51.5%)	20 (60.6%)	20 (60.6%)	0.689
	III	16 (48.5%)	13 (39.4%)	13 (39.4%)	
Operating time (min)		29.91 ± 5.39	27.06 ± 4.48	28.38 ± 5.09	0.070

### 3.2 Comparison of perioperative hemodynamics

There were no statistically significant differences in vital signs among the three groups at time points T0, T1, and T4 (*P* > 0.05).

Vital signs differed significantly among the three patient groups at T2 and T3 (*P* < 0.001). Post-hoc comparisons showed the R group had higher MAP and HR than RSL and PSL (*P* < 0.001). SpO_2_ was lower in the R group than in RSL and PSL (*P* < 0.001). MAP and HR did not differ significantly between RSL and PSL (*P* > 0.05), but SpO_2_ in RSL was higher than in PSL (*P* = 0.003; *P* = 0.004). See Table 2. To examine longitudinal changes in MAP, HR, and SpO_2_, we used linear mixed-effects models. Perioperative hemodynamic changes varied by group, as evidenced by significant time and group × time interaction effects. Consistent results emerged from sensitivity analyses using repeated-measures ANOVA. Detailed findings appear in Supplementary Table 1.

**TABLE 2:** Comparison of MAP, HR, and SpO_2_ values (*n* = 33).

	R	RSL	PSL	*F*	*P*-value
MAP (mmHg)					
T0	99.18 ± 9.53	102.50 ± 6.60	101.81 ± 9.31	1.391	0.254
T1	95.94 ± 8.89	97.58 ± 6.60	95.76 ± 8.47	0.511	0.601
T2	113.52 ± 6.05	102.00 ± 5.99	101.97 ± 6.28	39.269	<0.001
T3	111.15 ± 6.10	100.41 ± 6.09	100.56 ± 6.54	32.127	<0.001
T4	98.09 ± 4.56	95.85 ± 4.55	97.16 ± 4.59	2.033	0.137
HR (times/min)					
T0	80.88 ± 11.03	87.24 ± 11.82	85.61 ± 11.70	2.713	0.071
T1	87.64 ± 11.74	82.97 ± 11.26	89.27 ± 11.84	2.615	0.078
T2	106.61 ± 11.70	85.45 ± 11.21	92.24 ± 11.82	28.700	<0.001
T3	103.61 ± 11.70	84.42 ± 11.12	91.24 ± 11.82	23.386	<0.001
T4	86.67 ± 11.69	81.91 ± 11.12	88.24 ± 11.82	2.689	0.073
SpO2 (%)					
T0	92.88 ± 1.98	93.55 ± 2.77	92.82 ± 2.94	0.795	0.454
T1	96.64 ± 1.54	96.91 ± 1.65	96.03 ± 1.51	2.724	0.071
T2	89.33 ± 1.05	92.85 ± 1.25	91.85 ± 1.25	76.516	<0.001
T3	90.30 ± 1.05	92.76 ± 1.28	91.79 ± 1.27	34.947	<0.001
T4	92.64 ± 1.34	93.21 ± 1.19	92.70 ± 1.36	1.956	0.147

### 3.3 Comparison of cognitive function

There were no differences in preoperative MMSE, MoCA, SAS, and SDS scores among the three groups (*P* > 0.05).

On postoperative day 1, statistically significant differences in MMSE, MoCA, SAS, and SDS scores were observed among the three groups (*P* < 0.05), with the RSL group showing higher MMSE and MoCA scores and lower SAS and SDS scores than the R and PSL groups. No significant differences were observed between Groups R and PSL. By postoperative day 7, however, these differences were no longer statistically significant, indicating that the initial group differences diminished over time. See Table 3. MMSE, MoCA, SAS, and SDS scores were assessed longitudinally using linear mixed-effects models. All outcomes showed significant time and group × time interaction effects, but no significant overall group effects. Similar results emerged from sensitivity analyses conducted via repeated-measures ANOVA. For detailed findings, refer to Supplementary Table 2.

**TABLE 3:** Comparison of cognitive function assessments (*n* = 33).

Group	MMSE			MoCA			SAS			SDS		
	d0	d1	d7	d0	d1	d7	d0	d1	d7	d0	d1	d7
R	28.03 ± 1.24	24.12 ± 1.22	26.76 ± 0.97	27.03 ± 1.24	22.91 ± 1.57	26.09 ± 1.07	32.94 ± 1.84	37.12 ± 1.69	34.85 ± 1.54	33.94 ± 1.84	38.00 ± 1.89	35.24 ± 1.62
RSL	28.24 ± 1.20	25.15 ± 1.33	27.00 ± 1.39	27.24 ± 1.20	24.18 ± 1.26	26.58 ± 1.09	32.91 ± 2.11	35.52 ± 2.21	34.06 ± 2.14	33.91 ± 2.11	36.67 ± 2.23	34.61 ± 2.16
PSL	28.00 ± 1.39	24.09 ± 1.51	26.27 ± 1.33	27.03 ± 1.38	23.24 ± 1.30	26.42 ± 1.32	33.48 ± 1.79	36.85 ± 1.79	34.58 ± 1.56	34.48 ± 1.79	37.88 ± 1.75	35.12 ± 1.83
*F*	0.35	6.54	2.92	0.301	7.51	1.49	0.94	6.69	1.69	0.94	4.64	1.06
*P*-value	0.704	0.002	0.059	0.738	0.001	0.231	0.393	0.002	0.190	0.393	0.012	0.351

### 3.4 Comparison of serum inflammatory marker levels

There were no statistically significant differences in CRP levels among the three groups at T0 and T4 (*P* > 0.05). At T0, TNF-α and COR levels were also not significantly different among groups. However, at T4, post-hoc comparisons showed that TNF-α and COR levels in the R group were significantly higher than in the RSL and PSL groups (*P* < 0.05) (*P* < 0.05), whereas no significant differences were observed between Groups RSL and PSL. See Table 4.

**TABLE 4:** Comparison of serum inflammatory marker levels before and after surgery (*n* = 33).

	TNF-α (pg/ml)		CRP (mg/L)		COR (ug/dl)	
	T0	T4	T0	T4	T0	T4
R	6.60 ± 0.87	14.95 ± 1.99	8.50 ± 0.65	11.41 ± 0.98	7.25 ± 1.18	9.86 ± 1.40
RSL	6.67 ± 0.61	14.13 ± 1.47	8.41 ± 0.70	10.98 ± 0.79	7.82 ± 1.24	8.82 ± 1.53
PSL	6.57 ± 0.53	13.38 ± 0.87	8.45 ± 0.42	11.16 ± 0.24	7.48 ± 1.15	8.85 ± 1.55
*F*	0.18	8.75	0.20	2.77	1.92	5.16
*P*-value	0.84	<0.001	0.81	0.067	0.153	0.007

Linear mixed-effects models were used to analyze TNF-α, CRP, and COR levels over time. Significant time effects were observed for all three markers. Significant group × time interaction effects were observed for TNF-α and COR, but not for CRP. Repeated-measures ANOVA sensitivity analyses showed generally consistent results. Detailed results are presented in Supplementary Table 3.

### 3.5 Comparison of anesthetic effects

Group R had significantly higher remifentanil consumption than Groups RSL and PSL (*P* < 0.001), with no significant difference between the latter two groups (*P* = 0.312). Recovery time was significantly longer in the R group compared to the RSL group (*P* = 0.008). The PSL group’s recovery time was intermediate, but these differences were not statistically significant (all *P* > 0.05). Both RSL and PSL groups had reduced intraoperative vocal cord movement, resulting in higher patient satisfaction (*P* < 0.05). See Table 5.

**TABLE 5:** Comparison of anesthetic effects.

Variable	R	RSL	PSL	*P-value*
Vocal cord movement, *n* (%)				<0.001
Open	10 (30.3%)	28 (84.8%)	27 (81.8%)	
Active	20 (60.6%)	4 (12.1%)	5 (15.2%)	
Closed	3 (9.1%)	1 (3.0%)	1 (3.0%)	
Time to awakening (min)	10.00 ± 2.78	8.06 ± 2.38	9.36 ± 2.58	0.010
Patient satisfaction, *n* (%)				<0.001
Satisfied	18 (54.5%)	31 (93.9%)	30 (90.9%)	
Dissatisfied	15 (45.5%)	2 (6.1%)	3 (9.1%)	
Surgeon satisfaction, *n* (%)				0.015 <0.001
Satisfied	21 (63.6%)	30 (90.9%)	28 (84.8%)	
Dissatisfied	12 (36.4%)	3 (9.1%)	5 (15.2%)	
Total remifentanil dose (mg)	0.81 ± 0.13	0.55 ± 0.12	0.60 ± 0.15	

### 3.6 Incidence of perioperative adverse events

Comparisons of intraoperative coughing, hypoxemia, and postoperative nausea and vomiting incidence among the three groups revealed statistically significant differences (*P* < 0.05). Further pairwise comparisons revealed that the incidence of the aforementioned adverse events in the R group was significantly higher than that in the RSL and PSL groups (*P* < 0.05); the incidence of intraoperative hypoxemia in the RSL group was lower than that in the PSL group (*P* = 0.046); however, there were no statistically significant differences in the incidence of the remaining two adverse events between the RSL and PSL groups (*P* > 0.05). See Table 6.

**TABLE 6:** Incidence of adverse reactions [*n* = 33, cases (x/%)].

Group	Intraoperative coughing	Intraoperative hypoxemia	Postoperative nausea and vomiting
R	20 (60.6%)	17(51.5%)	10 (30.3%)
RSL	2 (6.1%)	1(3.0%)	3 (9.1%)
PSL	2 (6.1%)	6 (18.2%)	4 (12.1%)
*X* ^2^	35.64	22.11	6.11
*P*-value	<0.001	<0.001	0.047

## 4 Discussion

With the growing popularity of patient-centered care, demand for pain-free bronchoscopy is rising. Existing studies have demonstrated that general anesthesia provides more stable maintenance of vital signs, reduces the incidence of intraoperative adverse events, and enhances patient comfort and satisfaction. However, elderly patients with coronary heart disease constitute a special population; not only are they prone to severe hemodynamic fluctuations during the perioperative period, but they also have a high incidence of postoperative complications, such as cognitive decline. Therefore, developing an anesthetic regimen that provides effective sedation and analgesia while maximizing circulatory stability, minimizing the stress response, and preserving organ function is of significant clinical importance. This study compared three different anesthetic strategies to evaluate the clinical utility of remimazolam combined with ultrasound-guided superior laryngeal nerve block in this specific patient population.

### 4.1 The effects of combined therapies on perioperative hemodynamics

The primary outcomes showed that in the R group [patients who received remimazolam without superior laryngeal nerve block (SLNB)], blood pressure and heart rate increased significantly, and oxygen saturation decreased significantly when the bronchoscope passed through the vocal cords and at the procedure’s end. In contrast, vital signs in the RSL group (patients who received remimazolam plus SLNB) and PSL group (patients who received propofol plus SLNB) remained relatively stable at these times. Therefore, these results indicate that SLNB mitigates laryngopharyngeal irritation caused by bronchoscopy by blocking the internal branch of the superior laryngeal nerve, which anesthetizes the mucosal sensation in areas it innervates, such as the tongue root, epiglottis, and glottic cleft (4). Furthermore, previous studies have found that remimazolam, when used during bronchoscopy, can alleviate hypotension caused by propofol (5). However, in this study, there were no significant differences in HR or MAP between the RSL and PSL groups. From a pharmacological perspective, remimazolam’s inhibitory effects on the cardiovascular system–particularly on sympathetic nerve activity and vascular tone–are milder than those of propofol (6). Thus, for elderly patients with coronary heart disease and reduced myocardial reserve, this may help reduce the risk of post-induction hypotension.

### 4.2 The effect of combination therapy on early postoperative cognitive function

Elderly patients are prone to developing POCD following general anesthesia. The mechanism underlying this condition is complex and may be associated with multiple factors, including surgical trauma, inflammatory responses, anesthetic agents, and unstable cerebral perfusion during surgery (7). Recent randomized studies have emphasized the importance of optimizing anesthetic strategies in elderly patients to improve perioperative outcomes and reduce postoperative complications (8). Recent studies have also highlighted the potential contribution of perioperative neuroinflammation to postoperative cognitive dysfunction and delirium in elderly patients. Patumporn et al. conducted a meta-analysis of 20 clinical studies with POCD as the primary outcome, indicating that the onset of POCD within 30 days postoperatively leads to prolonged hospital stays and increased mortality risk (9). This study found that on the first postoperative day, patients in the RSL group had significantly higher cognitive function scores (Mini-Mental State Examination, Montreal Cognitive Assessment) than those in the R and PSL groups, while their anxiety and depression scores (Self-Rating Anxiety Scale, Self-Rating Depression Scale) were significantly lower than those in the other two groups. Although early studies generally suggested that benzodiazepines increase the risk of postoperative delirium, several recent studies have presented contrary opinions (10, 11). Regarding the outcomes of this study, we found that they may result from the combined effects of multiple factors. First, compared with the traditional midazolam, remimazolam has a shorter elimination half-life, does not accumulate in the body, and has a minimal impact on the central nervous system postoperatively. Second, SLNB provides effective analgesia and may reduce intraoperative stress and inflammation, as shown by the TNF-α and COR results in this study. While previous research indicates that strong inflammatory responses may disrupt the blood-brain barrier and cause neuronal dysfunction, these mechanisms were not evaluated in this study. Recent studies have further highlighted the potential contribution of perioperative neuroinflammation to postoperative delirium and cognitive dysfunction in elderly patients (12). Furthermore, the combined regimen ensures more stable hemodynamics, guaranteeing adequate and consistent cerebral perfusion and preventing cerebral ischemia-reperfusion injury that could result from severe blood pressure fluctuations. A study by Park et al. also found that patients who received remimazolam had higher postoperative cognitive function scores than those who received propofol (13). We speculate that this may be due to remimazolam providing more stable hemodynamics. By the 7th postoperative day, differences among the three groups had disappeared, indicating that these effects were transient. However, the combined regimen was associated with better early postoperative cognitive function outcomes in this study.

### 4.3 Effects of the combined protocol on the Body’s stress and inflammatory responses

Surgical trauma and intense stimulation can trigger stress and inflammatory responses in the body, manifested by increased release of pro-inflammatory factors such as TNF-α and elevated levels of stress hormones such as cortisol (14). These responses may be more pronounced in elderly patients and may affect postoperative recovery. In this study, TNF-α and cortisol levels in the R group were significantly higher than those in the RSL and PSL groups immediately postoperatively, further confirming the pivotal role of SLNB in suppressing noxious stimuli at the source and alleviating systemic stress responses. A domestic study reached the same conclusion: for patients undergoing painless bronchoscopy, the use of SLNB rather than topical anesthesia more effectively reduces stress responses (15). Furthermore, previous experimental studies have suggested that remimazolam may have anti-inflammatory and cardioprotective effects via mechanisms such as NLRP3/IL-1β regulation and reduced myocardial ischemia/reperfusion injury (16), which is of great significance for patients with coronary heart disease who have certain myocardial blood supply disorders. However, as this study did not assess mechanistic biomarkers or myocardial-specific injury markers, these mechanisms remain speculative. No intergroup differences in CRP levels were observed in this study; we consider this may be related to the kinetics of the CRP response. As a late-phase acute-phase reactant, CRP typically peaks 24–48 h after stimulation. Since this study collected blood samples only immediately postoperatively, it may have missed the peak of its expression. Future studies could extend the observation period to more comprehensively evaluate the long-term effects of different anesthetic regimens on the inflammatory response.

### 4.4 Analysis of anesthetic efficacy and drug dosage in the combined protocol

Our findings suggest that the two patient groups who received nerve blocks (RSL and PSL) demonstrated significantly better anesthetic outcomes across multiple indicators than the R group. SLNB precisely blocks the internal branch of the superior laryngeal nerve, reducing laryngeal sensation and suppressing airway reflexes during bronchoscopy. Adequate vocal cord opening and reduced intraoperative coughing provided endoscopists with an ideal field of view and access, which may explain the significant improvement in operator satisfaction. A domestic study found that, in painless bronchoscopy, remimazolam improved patient satisfaction more than propofol. In this study, both the RSL and PSL groups outperformed the R group in terms of patient satisfaction, though there was no significant difference between the two. We attribute this to the fact that patients in both groups underwent SLNB, which reduced surgical stress and, consequently, lowered their demand for intraoperative analgesics. Reduced drug dosage, particularly for opioids, can effectively shorten recovery time and lower the incidence of postoperative nausea and vomiting (17), thereby enhancing patient comfort and satisfaction. These findings fully demonstrate the advantages of “multimodal analgesia,” which achieves optimal anesthetic outcomes by combining anesthetic methods with different mechanisms of action, while simultaneously reducing the dosage of individual drugs and their associated side effects (18). The duration of recovery from general anesthesia is also a key indicator of anesthetic efficacy; prolonged recovery time can significantly increase the incidence of postoperative complications in elderly patients (19). Although there were no significant differences between the RSL and PSL groups in most anesthetic efficacy indicators, the RSL group had the shortest recovery time. This is attributable to the ultra-short duration of action of remimazolam. It should also be noted that BIS interpretation during remimazolam sedation is controversial, as BIS values under benzodiazepine-based anesthesia may not accurately reflect sedation depth compared to propofol. Additionally, in special circumstances, remimazolam has a specific antagonist, flumazenil, which can promptly terminate anesthesia. Furthermore, studies have found that the use of flumazenil for postoperative emergence from general anesthesia in elderly patients is associated with no adverse reactions (20).

### 4.5 Safety evaluation of the combined protocol

Safety is the primary criterion for evaluating the merits of an anesthetic protocol. The results of this study indicate that the use of superior laryngeal nerve block significantly reduces the incidence of intraoperative coughing, which is crucial for preventing examination interruptions, aspiration, and severe hemodynamic fluctuations. In elderly patients with coronary heart disease, reduced myocardial reserve capacity and diminished vascular elasticity make them more susceptible to exacerbation of myocardial ischemia and induction of arrhythmias due to severe hemodynamic fluctuations. Compared with traditional blind puncture, ultrasound-guided SLNB allows for clear identification of nerves and surrounding structures. In this study, no procedure-related complications, such as hematomas or nerve injury, were observed in patients who underwent SLNB. However, as an invasive procedure, we must still exercise caution and remain vigilant for signs of local anesthetic toxicity.

On the other hand, due to the lower remifentanil dose, the incidence of PONV was significantly lower in the PSL and RSL groups. Although previous studies have shown that propofol has some antiemetic effects (21), in our study, the incidence of PONV was similar between the PSL and RSL groups. A study by Cao et al. also noted that, when compared with remimazolam, propofol did not effectively reduce the incidence of PONV (22).

Because bronchoscopy procedures occupy the airway, intraoperative respiratory management is a major concern during anesthetic management. In terms of preventing hypoxemia, the RSL group performed particularly well; its incidence rate (3.0%) was significantly lower than that of the R group (51.5%) and the PSL group (18.2%). Additionally, the primary outcomes showed that patients in the RSL group had higher SpO_2_ levels at both T2 and T3 than those in the other two groups. The lower incidence of hypoxemia observed in the RSL group may be related to the combined effect of SLNB and remimazolam rather than to remimazolam alone. Effective airway sensory blockade may reduce procedural stimulation and decrease the need for supplemental sedatives and opioids, thereby improving respiratory safety. Propofol induces a deeper level of anesthesia, which can easily lead to apnea and decreased blood oxygen levels (23). A meta-analysis by Zhou et al. showed that the success rate of remimazolam in bronchoscopy sedation is comparable to that of traditional drugs, while its respiratory and circulatory depressant effects are weaker (24). A study by Zhang et al. also noted that, while preserving spontaneous breathing, the combination of remimazolam and alfentanil significantly reduced the incidence of respiratory depression compared to propofol, and decreased adverse reactions such as postoperative dizziness and abdominal distension (25). In this study, thanks to the effective analgesia provided by the superior laryngeal nerve block, the RSL group met procedural requirements without excessive sedation or analgesia, thereby maximizing the advantages of both approaches. In contrast, although the PSL group also received a nerve block, the strong respiratory depression associated with propofol itself kept the risk of hypoxemia at a relatively high level.

### 4.6 Limitations of this study

This study has several limitations. First, it was a single-center study with a small sample, raising the possibility of selection bias. Larger multicenter randomized controlled trials are necessary. Second, while inflammatory and stress-related biomarkers were examined, myocardial-specific injury and mechanistic biomarkers were omitted, and confounding factors were not fully controlled. Broader biomarker assessments and stricter control for comorbidities should be applied in future studies. Third, capnography was not used routinely, which may have restricted respiratory monitoring and hindered early detection of depression. Fourth, BIS values during remimazolam sedation may not correspond to sedation depth achieved under propofol-based anesthesia; our relatively deep BIS target range may limit generalizability. Fifth, cognitive assessment relied solely on MMSE and MoCA within 7 days postoperatively, without formal diagnostic evaluation for neurocognitive disorders–repeated testing may have induced learning effects. Sixth, we assessed adverse events using only clinical observation during the perioperative period, without formal severity grading or long-term follow-up. No nerve block complications occurred, but the small sample size prevents conclusions about SLNB safety. Future studies should use larger samples and comprehensive safety monitoring. Finally, complete blinding was infeasible due to the intervention, which could introduce bias. Future studies should employ more rigorous blinding and longer-term outcome assessments.

## 5 Conclusion

Ultrasound-guided superior laryngeal nerve block combined with remimazolam may be a feasible anesthetic strategy for bronchoscopy in elderly patients with coronary heart disease. In this study, the regimen was associated with more stable perioperative hemodynamics and lower remifentanil consumption. It also resulted in improved glottic opening conditions, fewer respiratory adverse events, and higher patient and endoscopist satisfaction. Beyond these primary findings, the approach was also associated with lower postoperative TNF-α and COR levels and better early postoperative cognitive screening scores; however, these secondary findings should be interpreted as exploratory. As such, further multicenter studies with larger sample sizes and longer follow-up are needed to confirm these findings.

## Data Availability

The original contributions presented in this study are included in the article/Supplementary material, further inquiries can be directed to the corresponding author.
