# Supplementary material for: Clinical study of remimazolam combined with ultrasound-guided superior laryngeal nerve block in elderly patients with coronary heart disease undergoing bronchoscopy
Source: Front Med (Lausanne). 2026 Jul 30;13:1840825. doi: 10.3389/fmed.2026.1840825 (PMC13469081; doi:10.3389/fmed.2026.1840825)
Supplement: Supplementary file 1 [file Data_Sheet_1.pdf]

Supplementary Table S1. Linear mixed-effects model and repeated-measures ANOVA analyses of perioperative hemodynamic variables

Outcome	Model	Effect	df (df1, df2)	Statistic	P value	Effect size
MAP	LMM	Group	2	χ^2=4.473	0.107	—
MAP	LMM	Time	4	χ^2=407.961	<0.001	—
MAP	LMM	Group × Time	8	χ^2=186.744	<0.001	—
MAP	RM-ANOVA	Group	(2, 96)	F=6.076	0.003	0.112
MAP	RM-ANOVA	Time	(1.214, 116.567)	F=77.927	<0.001	0.448
MAP	RM-ANOVA	Group × Time	(2.428, 116.567)	F=22.636	<0.001	0.320
HR	LMM	Group	2	χ^2=5.559	0.062	—
HR	LMM	Time	4	χ^2=335363.711	<0.001	—
HR	LMM	Group × Time	8	χ^2=150489.461	<0.001	—
HR	RM-ANOVA	Group	(2, 96)	F=7.422	0.001	0.134
HR	RM-ANOVA	Time	(1.002, 96.155)	F=50848.957	<0.001	0.998
HR	RM-ANOVA	Group × Time	(2.003, 96.155)	F=18242.130	<0.001	0.997
SpO_2	LMM	Group	2	χ^2=4.029	0.133	—
SpO_2	LMM	Time	4	χ^2=455.173	<0.001	—
SpO_2	LMM	Group × Time	8	χ^2=70.063	<0.001	—
SpO_2	RM-ANOVA	Group	(2, 96)	F=22.194	<0.001	0.316
SpO_2	RM-ANOVA	Time	(2.542, 244.012)	F=176.572	<0.001	0.648
SpO_2	RM-ANOVA	Group × Time	(5.084, 244.012)	F=8.492	<0.001	0.150

Data are presented as mean ± SD. P values represent between-group comparisons at each time point. Detailed LMM primary analyses and RM-ANOVA sensitivity analyses are provided in Supplementary Table S1.
