# Supplementary material for: Clinical study of remimazolam combined with ultrasound-guided superior laryngeal nerve block in elderly patients with coronary heart disease undergoing bronchoscopy
Source: Front Med (Lausanne). 2026 Jul 30;13:1840825. doi: 10.3389/fmed.2026.1840825 (PMC13469081; doi:10.3389/fmed.2026.1840825)
Supplement: Supplementary file 2 [file Data_Sheet_2.pdf]

Supplementary Table S2. Linear mixed-effects model and repeated-measures ANOVA analyses of cognitive and emotional scores

Outcome	Model	Effect	df (df1, df2)	Statistic	P value	Effect size
MMSE	LMM	Group	2	χ^2=0.709	0.701	—
MMSE	LMM	Time	2	χ^2=939.554	<0.001	—
MMSE	LMM	Group × Time	4	χ^2=36.997	<0.001	—
MMSE	RM-ANOVA	Group	(2, 96)	F=2.719	0.071	0.054
MMSE	RM-ANOVA	Time	(1.830, 175.672)	F=1196.669	<0.001	0.926
MMSE	RM-ANOVA	Group × Time	(3.660, 175.672)	F=8.969	<0.001	0.157
MoCA	LMM	Group	2	χ^2=0.625	0.732	—
MoCA	LMM	Time	2	χ^2=979.497	<0.001	—
MoCA	LMM	Group × Time	4	χ^2=42.004	<0.001	—
MoCA	RM-ANOVA	Group	(2, 96)	F=2.552	0.083	0.050
MoCA	RM-ANOVA	Time	(1.651, 158.487)	F=1286.871	<0.001	0.931
MoCA	RM-ANOVA	Group × Time	(3.302, 158.487)	F=10.183	<0.001	0.175
SAS	LMM	Group	2	χ^2=2.052	0.358	—
SAS	LMM	Time	2	χ^2=462.924	<0.001	—
SAS	LMM	Group × Time	4	χ^2=55.125	<0.001	—
SAS	RM-ANOVA	Group	(2, 96)	F=2.249	0.111	0.045
SAS	RM-ANOVA	Time	(1.869, 179.434)	F=661.766	<0.001	0.873
SAS	RM-ANOVA	Group × Time	(3.738, 179.434)	F=13.364	<0.001	0.218
SDS	LMM	Group	2	χ^2=1.932	0.381	—
SDS	LMM	Time	2	χ^2=576.330	<0.001	—
SDS	LMM	Group × Time	4	χ^2=42.639	<0.001	—
SDS	RM-ANOVA	Group	(2, 96)	F=1.668	0.194	0.034
SDS	RM-ANOVA	Time	(1.823, 175.020)	F=804.327	<0.001	0.893
SDS	RM-ANOVA	Group × Time	(3.646, 175.020)	F=10.337	<0.001	0.177

Data are presented as mean ± SD. P values represent between-group comparisons at each time point. Detailed LMM primary analyses and RM-ANOVA sensitivity analyses are provided in Supplementary Table S2.
