# Supplementary material for: Clinical study of remimazolam combined with ultrasound-guided superior laryngeal nerve block in elderly patients with coronary heart disease undergoing bronchoscopy
Source: Front Med (Lausanne). 2026 Jul 30;13:1840825. doi: 10.3389/fmed.2026.1840825 (PMC13469081; doi:10.3389/fmed.2026.1840825)
Supplement: Supplementary file 3 [file Data_Sheet_3.pdf]

Supplementary Table S3. Linear mixed-effects model and repeated-measures ANOVA analyses of serum inflammatory and stress markers

Outcome	Model	Effect	df (df1, df2)	Statistic	P value	Effect size
TNF-α	LMM	Group	2	χ^2=0.010	0.995	—
TNF-α	LMM	Time	1	χ^2=837.911	<0.001	—
TNF-α	LMM	Group × Time	2	χ^2=14.184	<0.001	—
TNF-α	RM-ANOVA	Group	(2, 96)	F=7.449	<0.001	0.134
TNF-α	RM-ANOVA	Time	(1, 96)	F=2065.356	<0.001	0.956
TNF-α	RM-ANOVA	Group × Time	(2, 96)	F=7.092	0.001	0.129
CRP	LMM	Group	2	χ^2=0.330	0.848	—
CRP	LMM	Time	1	χ^2=812.036	<0.001	—
CRP	LMM	Group × Time	2	χ^2=5.371	0.068	—
CRP	RM-ANOVA	Group	(2, 96)	F=1.537	0.220	0.031
CRP	RM-ANOVA	Time	(1, 96)	F=2151.609	<0.001	0.957
CRP	RM-ANOVA	Group × Time	(2, 96)	F=2.685	0.073	0.053
COR	LMM	Group	2	χ^2=2.969	0.227	—
COR	LMM	Time	1	χ^2=66.491	<0.001	—
COR	LMM	Group × Time	2	χ^2=13.889	<0.001	—
COR	RM-ANOVA	Group	(2, 96)	F=1.291	0.280	0.026
COR	RM-ANOVA	Time	(1, 96)	F=80.661	<0.001	0.457
COR	RM-ANOVA	Group × Time	(2, 96)	F=6.945	0.002	0.126

Data are presented as mean ± SD. P values represent between-group comparisons at each time point. Detailed LMM primary analyses and RM-ANOVA sensitivity analyses are provided in Supplementary Table S3.
